# The Registration of Deaths

**Published:** 1902-03

**Authors:** 


The Registration of Deaths.—The Census Bureau, realizing the imperative necessity for a uniform system of vital statistics, is making an intelligent and earnest effort to bring about such uniformity. But a great obstacle is found in the fact that some States have no law requiring the registration of births and deaths at all; there is, in those States no way of proving, should it be necessary to prove, that any citizen ever was born, or ever died. Texas is prominent amongst these benighted States. She "has a system of vital statistics for *pigs,* but none for people" (Smith). This obstacle must therefore be overcome before the census people talk about uniformity. This they will seek to do, as will be seen by reading Dr. King's letter herewith published, by "bringing the subject to the attention of Governors and Legislatures." I wish them better success than we doctors and the Red-Back have had; for we have been urging, for twenty years, the creation of a Bureau of Vital and Mortuary Statistics, as a basis for sanitary reform legislation. The Journal has preached it in season and out, but it had as well sung psalms to a dead horse. I regret that I have not room to print the "paper of the committee" referred to by Dr. King. Interested persons may get a copy by simply asking for it.

WASHINGTON, D. C., February 14, 1902.

*Dr. F. E. Daniel, Editor Texas Medical Journal, Austin, Texas.*

SIR: I have the honor and pleasure to invite your personal attention to the enclosed Census Circular No. 71, on the registration of deaths, in the earnest expectation of enlisting your active interest in the matter.

You will find in the circular an account of the effort of the Census Office to extend the registration of deaths; the widespread interest that has been aroused; the practical results attained in the way of uniformity and quality of data; the co-operation now being given by the American Public Health Association; and, also a paper prepared by a committee of that body stating the essentials of a registration law, which bears the endorsement of the Census Office and the Commissioner of Labor, and which, too, contains model forms of death and burial permit certificates.

This circular is designed by the Census Office and the American Public Health Association to give the necessary additional and official impetus to the extention of the registration of deaths into the cities and States that are now non-registration areas, and also is intended to furnish the authorities of States and cities possessing such systems the most authoritative information to guide them toward the goal of national uniformity of systems, methods and results.

It will be brought by the Director of the Census to the immediate attention of the Governor and Legislatures of the non-registration States, and every effort will be made to procure the introduction and passage of the desired laws. In this direction your journal can render a great service to the government, humanity and science by exerting its powerful influence toward increasing public and professional interest in the present movement. Reproduction of such parts of the circular, particularly of the committee's paper, together with such editorial discussion as your columns will permit, will certainly fill the measure of my desire and be gratefully appreciated. Be good enough, also, to send me a copy of the issue in which the matter appears.

Thanking you, I remain,

Very respectfully,

W. A. KING, Chief Statistician.

